# Xenon in the treatment of panic disorder: an open label study

**DOI:** 10.1186/s12967-017-1237-1

**Published:** 2017-06-13

**Authors:** Alexander Dobrovolsky, Thomas E. Ichim, Daqing Ma, Santosh Kesari, Vladimir Bogin

**Affiliations:** 1 Pirogov Russian National Research Medical University, Moscow, Russia; 2 Institute of Mental Health and Addictology, Moscow, Russia; 3 Nobilis Therapeutics Inc, San Diego, CA USA; 4 Section of Anaesthetics, Pain Medicine and Intensive Care, Department of Surgery and Cancer, Faculty of Medicine, Imperial College London, Chelsea and Westminster Hospital, London, UK; 5 Department of Translational Neuro-Oncology and Neurotherapeutics, John Wayne Cancer Institute, Pacific Neuroscience Institute, Providence Saint John’s Health Center, Santa Monica, CA USA

**Keywords:** Panic disorder, Xenon therapy, Inhalation of xenon, Comorbidity

## Abstract

**Background:**

Current treatments of panic disorder (PD) are limited by adverse effects, poor efficacy, and need for chronic administration. The established safety profile of subanesthetic concentrations of xenon gas, which is known to act as a glutamate subtype NMDA receptor antagonist, coupled with preclinical studies demonstrating its effects in other anxiety related conditions, prompted us to evaluate its feasibility and efficacy in treatment of patients with PD.

**Methods:**

An open-label clinical trial of xenon–oxygen mixture was conducted in 81 patients with PD; group 1 consisting of patients only with PD (N = 42); and group 2 patients with PD and other comorbidities (N = 39).

**Results:**

Based on the analysis of the results of a number of psychometric scales used in this study (SAS, HADS, CGI), several conclusions can be made: (1) xenon is a potentially effective modality in acute treatment of PD; (2) an anti-panic effect of xenon administration persists for at least 6 months after the completion of the active phase of treatment; (3) xenon inhalation is well tolerated, with the drop-out rates being much lower than that of conventional pharmacotherapy (5.8% vs. 15%); (4) the severity of depressive disorders that frequently accompany PD can be significantly reduced with the use of xenon; (5) xenon may be considered as an alternative to benzodiazepines in conjunction with cognitive-behavioral therapy as a safe modality in treatment of anxiety disorder.

**Conclusions:**

These data support the need for randomized double-blind clinical trials to further study xenon-based interventions.

*Trial registration* This clinical trial was retrospectively registered on April 14th, 2017 as ISRCTN15184285 in the ISRCTN database.

## Background

One of the most common anxiety disorders is panic disorder (PD), with a 12 month prevalence in the US and in Europe estimated at 1.8 and 2.7% of the population, respectively [1, 2]; the main clinical feature of which is an unexpected panic attack (PA) that arises in the absence of any situational or emotional triggers, reaching its peak intensity within minutes, that is manifested by intense physical and cognitive symptoms, such as fear of recurrence, general health concerns, and behavioral changes [3, 4]. In addition to spontaneous PA, its other forms include situationally predisposed PA, “symptomatically mild” (“minor”) PA [5], in which less than 4 out of 13 symptoms listed in the DSM-IV are present, and “nocturnal” panic attacks that occur during phase 2 of the sleep cycle [6].

Panic disorder in its “pure” form is found only in 24.6% of cases, in 36.7% of cases it is accompanied by a comorbid disorder, in 13.3% —by 2, and in 23.5%—by 3 or more mental disorders, mainly anxiety and diseases of depressive spectrum [7]. Lecrubier et al. had shown that individuals with isolated panic attacks are more prone to the development of depression (45.6%) than to development of a panic disorder [8].

To date, the greatest clinical evidence of efficacy in the treatment of PD has been demonstrated with the use of selective serotonin reuptake inhibitors (SSRIs), serotonin–norepinephrine reuptake inhibitors (SNRIs) (drugs of first choice) and benzodiazepine tranquilizers (drugs of second choice) [9]. Disadvantages of SSRIs/SNRIs therapy include delayed onset of therapeutic effect (2–6 weeks), and side effects at the start of therapy, which can limit its use in the treatment of PD, especially given the importance of achievement of rapid anxiolytic effect [10, 11]. While benzodiazepines have immediate onset of action, their side effect profile is significant and includes excessive sedation, slow reaction time, dizziness, and possible paradoxical reactions such as anxiety [12]. In addition, knowledge of benzodiazepines’ high risk of dependence [13, 14], often forces patients with PD leading an active lifestyle, to seek other, alternative methods of treatment. These side effects in themselves can exacerbate PD and become the triggers of panic attacks. Historically, 18% of patients receiving SSRIs and 15% of patients receiving benzodiazepines drop-out from clinical studies [8]. To our knowledge, the contemporary scientific literature contains practically no data on the treatment of refractory PD, where both first- and second-line treatment options are ineffective. Additionally, there are frequent clinical scenarios where PD exists concomitantly with other psychiatric comorbidities or where panic attacks don’t reach the diagnostic threshold of a panic disorder, but nevertheless have a significant impact on the course of the underlying disease and impair social functioning. Thus, the search for an alternative treatment modality with non-habit forming anxiolytic effect and minimal side effects aimed at rapid relief of panic attacks represents an urgent unmet need in the treatment of PD.

Anxiety symptoms can occur in a variety of mental and substance abuse disorders. In particular, “neurovegetative” (poor sleep, sweating, loss of appetite, tremors, high blood pressure), anxiety and depressive symptoms are well established components of opioid and alcohol dependence and withdrawal, which are currently being treated with psychotropic drugs, including benzodiazepines, valproate and antiadrenergic agents [8, 15].

Xenon is a monatomic inert gas with very low chemical reactivity. It is colorless, odorless and heavy. Xenon has a very low blood-gas partition coefficient, rapidly penetrates the blood–brain barrier, which makes it an ideal general anesthetic. Xenon is a competitive *N*-methyl-d-aspartate (NMDA) receptor antagonist, which it exhibits through binding to glycine site of glutamatergic NMDA receptor [16]. In addition, xenon reduces excitatory neurotransmission through downregulation of 5-HT3 [17], nicotinic acetylcholine [18], potassium channel [19], HCN channel [20], and AMPA [21]. It also increases inhibitory neurotransmission by upregulating TREK1 [22]. Of relevance to fear associated conditions such as PD, the role of NMDA receptors in modulation of fear memories has previously been suggested [23]. Accordingly, Meloni et al. demonstrated that administration of xenon gas in a rat model of fear memory reconsolidation—a state in which recalled memories become susceptible to modification, reduced conditioned fear induced freezing [24].

Other psychiatric uses of xenon have been explored, for example, promising results on the use of inhaled xenon in opioid and alcohol withdrawal states, based on its pharmacokinetic effects have been reported [25–28]; in particular, its anti-stress properties, decreased sensitivity to pain and improved adaptation [29, 30]. However, there is paucity of research on the use of xenon outside of anesthesiology and addiction. According to some authors, its therapeutic properties are likely based on its effect on the glutamatergic system neuromodulation [31] via inhibition of NMDA receptors and reduction in binding of glutamate [31–33]. It was also shown that xenon at a dose of 30–50–70% in the gas mixture does not alter plasma concentrations of dopamine and norepinephrine, but causes a significant reduction of the level of adrenaline and cortisol [34].

It should also be noted that due to the biochemical inertness xenon, it exhibits no acute or chronic toxicity [35], embryotoxic or teratogenic effects, it is non-allergenic [18, 33], and does not alter the integrity of brain structures [36].

In recent studies it was demonstrated that the glutamatergic system plays a significant role in the regulation of anxiety. In particular, blockade of glutamatergic transmission in the periaqueductal gray matter lead to restoration of normal behavior in animals, and glutamate antagonists exhibited anxiolytic properties in experimental conditions [37].

In addition, preclinical studies have shown that blocking the glycine site NMDA-glutamate receptors results in anxiolytic effects [38]. Some supporting evidence that implicates glutamate in the pathogenesis of anxiety disorders stems from the efficacy of pregabalin, in which the mechanism of action is associated with inhibition of glutamate release [39]. Thus, on the basis of clinical data previously obtained from the use of xenon in anesthesiology and addiction medicine, as well as based on its receptor activity profile (reduction of glutamatergic neurotransmission) it can be expected that xenon possesses an independent anti-anxiety effect. The preliminary experience of using xenon in the outpatient treatment of various psychiatric and addictive diseases has been marked by its clear anxiolytic effect, which triggered our desire to study xenon’s effect on specific anxiety disorders. PD was selected because of its paroxysmal, easily quantifiable nature and a high degree of recurrence, and also because the “panic attack” phenomenon occurs widely in other anxiety states.

The clinical study presented aimed to: (a) study efficacy and adverse effect profile of xenon in acute treatment as a monotherapy for “pure” PD; (b) assess efficacy and adverse effect profile of xenon in treatment of PD in the presence of other mental illness comorbidities; and (c) quantify the duration of xenon’s therapeutic effect.

## Methods

### Patients

This investigator-initiated study was performed under a prospective clinical trial protocol approved by the Institute of Mental Health and Addictology, which is accredited by the Ministry of Health of the Russian Federation to conduct clinical trials (#57689). Study conduct was in compliance with all ethical standards and good clinical practice. All study participants provided written informed consent prior to undergoing any protocol-related procedures. The study was registered at http://www.isrctn.com (number in process, application #32439). Ninety outpatients with a diagnosis of “panic disorder” (F41.0) according to ICD-10 were enrolled through the Institute of Mental Health and Addictology. Five patients dropped out of the study due to minor side effects, predominantly lightheadedness and headaches, and 4 patients dropped out of the study for unspecified reasons. As the intention to treat analysis was not utilized due to the open label design of the study, 81 patients with PD (49 women and 32 men), mean age was 35.2 years (range 18–69) were studied. Patients were randomized into 2 groups: with “pure” PD (group 1) and “comorbid” PD, when it was co-diagnosed with other mental illnesses (group 2). All patients with isolated PD (group 1, n = 42) received monotherapy with xenon at the aforementioned schedule, while the majority of patients (94.9%) with PD and comorbid conditions, which were predominantly depression (group 2, n = 39), in addition to xenon administration continued treatment for comorbid psychiatric disorders, which mainly consisted of antidepressants (SSRIs and SNRIs). In these patients, the reason for xenon treatment was the increase in the frequency and severity of panic attacks despite ongoing treatment with stable pharmacotherapy of at least 3–6 months’ duration.

### Xenon administration

Administration of xenon was performed through inhalation of xenon–oxygen mixtures that were escalated from 15%/85% to 30%/70% with titration increments of 5% per session. Each patient in the study underwent between 6 and 7 treatments with xenon–oxygen mixture. The first three sessions were carried out daily and from session 4 onward—every other day. The selected dosing regime and the composition of the gas mixtures were based on the historical evidence of safety of subanesthetic use of xenon in imaging [40–42].

Medical grade xenon (“medksenon”^®^, 99.9999%, manufacturer: Atommedcenter, Moscow, Russia) and medical grade oxygen in separate containers were admixed. Mixing and administration of gases in preset concentration and volume was accomplished with the use of the medical device MAGi-AMTS1, which enables the operator to adjust the concentration of xenon in the gas mixture, and which contains the electronic flow meter with a software module that allows for such adjustments. Administration of xenon–oxygen mixture to the patient was carried out via a face mask. Patients were asked to slowly inhale, holding breath for 5–10 s; exhale into the loop and after 35–40 s exhale outside the contour and breath in the new portion of gas mixture. Xenon inhalation lasted from 2.5 to 4 min, and the xenon consumption was capped at 3.0 L per procedure. The patients were assessed subjectively by the provider, while the vital signs (pulse, blood pressure, oxygen saturation) were continuously monitored.

### Patient assessment

Patients were evaluated after each xenon inhalation and at 30 and 180 days after completion of treatment. To this end, we employed clinical psychopathological and clinical catamnestic methods, and psychometric scales that are widely used internationally to assess the treatment of mental disorders. Scale Assessment, Zung Self-Rating Anxiety Scale (SAS) was performed prior to starting therapy (V1), and at 1 and 6 months after treatment. According to this scale, SAS index of less than 45 points corresponds to the normal value, 45–59—to mild-to-moderate degree of anxiety, 60–74—to high degree of anxiety, more than 75—to an extremely high-level of anxiety. Hospital Anxiety and Depression Scale (Hospital Anxiety and Depression Scale, HADS_T-anxiety subscale, HADS_D-Depression subscale) was used prior to the (V1), after the third (V3) and sixth (V6) xenon administrations. Categories for the assessment for each of the following subscales are as follows: 0–7 points—normal (absence of reliable pronounced symptoms of anxiety/depression); 8–10 points—subclinical anxiety/depression; 11 points and above—symptomatic anxiety/depression. Clinical Global Impression Scale (CGI-I—improvement subscale, CGI-S—severity of the disease subscale) was used before treatment and after each of the following 6 xenon treatments (V1, V2, V3, V4, V5, V6).

Statistical analysis of the results was carried out via statistical and analytical methods using Microsoft Excell 2000 program and with Statistica statistical tools (http://www.statsoft.com/, http://www.statsoft.ru/).

## Results

The two groups of patients, with “pure” PD (group 1, n = 42) and with “comorbid” PD (group 2, n = 39) were well matched (Table 1). For patients in group 2 the following comorbid disorders were most commonly observed: mixed anxiety-depressive disorder (43.6%), bipolar affective disorder (10.3%), recurrent depressive disorder (10.3%), obsessive–compulsive disorder (5.1%), and other nonpsychotic mental disorders (12.8%, heading F48).

**Table 1:** Social and demographic characteristics of the patients

	Group		
	“Pure” PD (n = 42)	“Comorbid” PD (n = 39)	Total (n = 81)
Age, years			
Mean	36.1	34.3	35.2
Standard deviation	12.90	12.20	12.52
Median	32.0	33.0	33.0
Minimum	19	18	18
Maximum	69	68	69
Sex			
Male, n (%)	22 (52.4%)	10 (25.6%)	32 (39.5%)
Female, n (%)	20 (47.6%)	29 (74.4%)	49 (60.5%)
Employment			
No, n (%)	19 (45.2%)	18 (46.2%)	37 (45.7%)
Yes, n (%)	23 (54.8%)	21 (53.8%)	44 (54.3%)
Disease duration, months			
Mean	8.9	16.9	12.8
Standard deviation	5.28	7.54	7.60
Median	6.0	18.0	12.0
Minimum	3	3	3
Maximum	18	24	24
Marriage status			
No, n (%)	16 (38.1%)	21 (53.8%)	37 (45.7%)
Yes, n (%)	26 (61.9%)	18 (46.2%)	44 (54.3%)
Children			
No, n (%)	17 (40.5%)	21 (53.8%)	38 (46.9%)
Yes, n (%)	25 (59.5%)	18 (46.2%)	43 (53.1%)

**Fig. 1:**
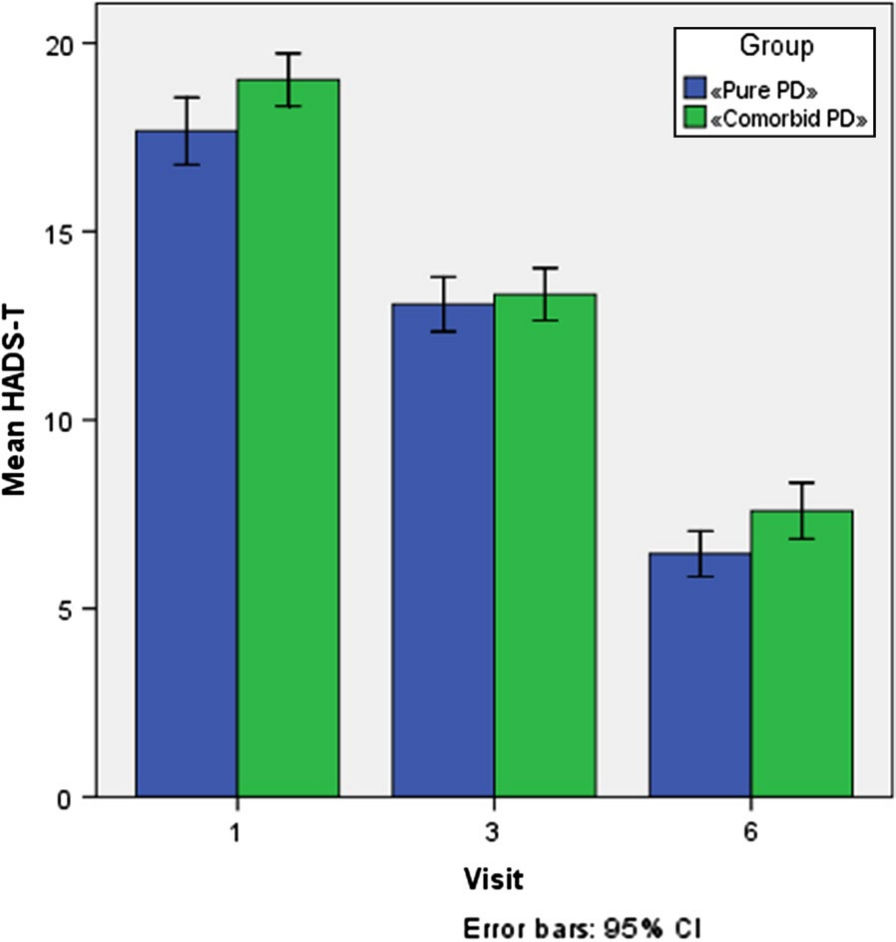
Reduction in Anxiety Score on the HADS_T Scale after Xenon Administration. Patients with only PD (group 1, n = 42) and “comorbid” PD (group 2, n = 39) where administered 6–7 sessions of xenon inhalation as described in “Methods”. Analysis of HADS_T score was performed. *Error bars* indicate 95% CI

Changes in the subscale of “anxiety” in Hospital Anxiety and Depression Scale (HADS_T) are presented in Fig. 1. The total score on this scale in both groups corresponded to the level of “clinically severe anxiety” (17.7 and 19.0, respectively), and showed a decrease (−4.6 and 5.7 points, respectively) after 3 sessions (V3) of xenon administration (13.3 and 13.3, respectively). By the end of treatment (V6), the overall scores in both groups corresponded to the category of the “norm” for HADS_T Scale. Statistical analysis of the changes in SAS Scale using paired test samples is presented in Table 2.

**Table 2:** Results of statistical analysis of HADS_T assessments of changes from baseline (V1) using a paired t test for the evaluation visits within each patient group

	Paired differences					t	df	Sig. (2-tailed)
	Mean	Std. deviation	Std. error mean	95% confidence interval				
				Lower	Upper			
Group 1								
HADS-T, V3 to HADS-T, V1	−4.595	3.379	.521	−5.648	−3.542	−8.813	41	.000
HADS-T, V6 to HADS-T, V1	−11.214	3.440	.531	−12.286	−10.142	−21.129	41	.000
Group 2								
HADS-T, V3 to HADS-T, V1	−5.692	1.749	.280	−6.259	−5.125	−20.319	38	.000
HADS-T, V6 to HADS-T, V1	−11.436	2.882	.461	−12.370	−10.502	−24.782	38	.000

Analysis of Clinical Global Impression Scale Improvement Subscale (CGI-I) changes after the third treatment shows a more significant improvement with xenon treatment (“marked improvement” on the CGI-I) in group 1 than in group 2 (40.5 and 10.3%, respectively, when compared to baseline). This trend persisted after 6 treatments: the indicator “very much improved” in patients with “pure” PD was 52.4%, while for those with “comorbid” PD it was only 12.8% (Table 3). According to the Clinical Global Impression Scale Severity of the Disease Subscale (CGI-S) (Table 4), before the start of treatment, both groups of patients demonstrated a pronounced degree of impairment: the indicator “significantly pronounced disease” was at 90.5 and 87.2%, respectively. After the third procedure, reduction in the severity of disorders was more pronounced in group 1: the indicator “moderately severe disease” was 48.7 and 11.9%, respectively. At the same time, upon completion of xenon treatments the differences between the two groups disappeared and most patients in both groups reached the “borderline” level (82.1 and 88.1%, respectively).

**Table 3:** Changes in CGI-I Scale during treatment

	Group					
	“Pure” PD (n = 42)		“Comorbid” PD (n = 39)		Total (n = 81)	
	n	%	n	%	n	%
CGI-I, V2						
Marked improvement	5	11.9	5	12.8	10	12.3
Minimal improvement	20	47.6	24	61.5	44	54.3
No changes	15	35.7	10	25.6	25	30.9
Minimal deterioration	1	2.4	0	.0	1	1.2
Marked deterioration	1	2.4	0	.0	1	1.2
Overall	42	100.0	39	100.0	81	100.0
CGI-I, V3						
Marked improvement	17	40.5	4	10.3	21	25.9
Minimal improvement	23	54.8	27	69.2	50	61.7
No changes	2	4.8	8	20.5	10	12.3
Overall	42	100.0	39	100.0	81	100.0
CGI-I, V4						
Marked improvement	34	81.0	10	25.6	44	54.3
Minimal improvement	8	19.0	25	64.1	33	40.7
No changes	0	.0	4	10.3	4	4.9
Overall	42	100.0	39	100.0	81	100.0
CGI-I, V5						
Very marked improvement	2	4.8	0	.0	2	2.5
Marked improvement	40	95.2	24	61.5	64	79.0
Minimal improvement	0	.0	15	38.5	15	18.5
Overall	42	100.0	39	100.0	81	100.0
CGI-I, V6						
Very marked improvement	22	52.4	5	12.8	27	33.3
Marked improvement	20	47.6	34	87.2	54	66.7
Overall	42	100.0	39	100.0	81	100.0

**Table 4:** Changes in CGI-S Scale during treatment

	Group					
	“Pure ”PD (n = 42)		“Comorbid” PD (n = 39)		Total (n = 81)	
	n	%	n	%	n	%
CGI-S, V1						
Moderately expressed disease	4	10.3	1	2.4	5	6.2
Significantly expressed disease	34	87.2	38	90.5	72	88.9
Serious disease	1	2.6	3	7.1	4	4.9
Total	39	100.0	42	100.0	81	100.0
CGI-S, V2						
Moderately expressed disease	19	48.7	1	2.4	20	24.7
Significantly expressed disease	20	51.3	39	92.9	59	72.8
Serious disease	0	.0	2	4.8	2	2.5
Total	39	100.0	42	100.0	81	100.0
CGI-S, V3						
Moderately expressed disease	19	48.7	5	11.9	24	29.6
Significantly expressed disease	20	51.3	37	88.1	57	70.4
Total	39	100.0	42	100.0	81	100.0
CGI-S, V4						
Weakly expressed disease	0	.0	20	47.6	20	24.7
Moderately expressed disease	20	51.3	22	52.4	42	51.9
Significantly expressed disease	19	48.7	0	.0	19	23.5
Total	39	100.0	42	100.0	81	100.0
CGI-S, V5						
Borderline state	0	.0	6	14.3	6	7.4
Weakly expressed disease	1	2.6	36	85.7	37	45.7
Moderately expressed disease	37	94.9	0	.0	37	45.7
Significantly expressed disease	1	2.6	0	.0	1	1.2
Total	39	100.0	42	100.0	81	100.0
CGI-S, V6						
Normal state	0	.0	5	11.9	5	6.2
Borderline state	32	82.1	37	88.1	69	85.2
Weakly expressed disease	7	17.9	0	.0	7	8.6
Total	39	100.0	42	100.0	81	100.0

Thus, by analyzing the changes in the indices of psychometric scales (HADS_T, CGI-I, CGI-S) it can be concluded that the use of xenon treatment in PD produced rapid onset of action, statistically significant clinical improvement, and complete cessation of panic attacks after the 6th treatment.

**Fig. 2:**
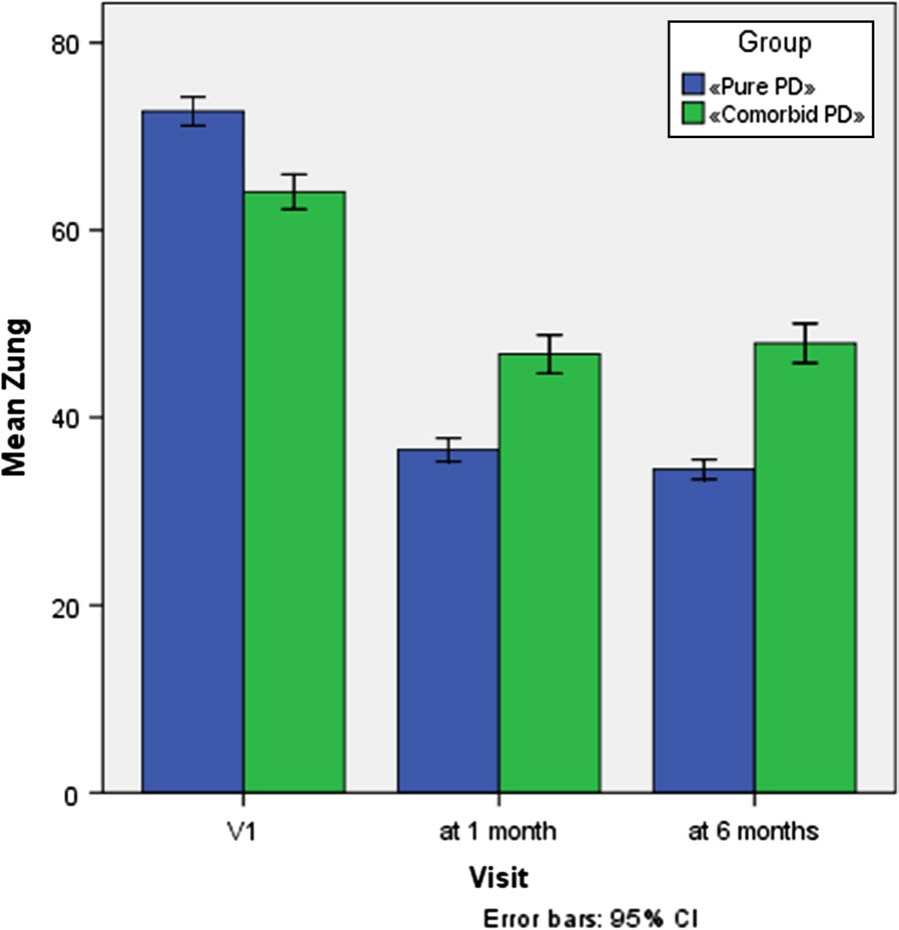
Reduction of the Zung Self-Rating Anxiety Scale (SAS) Score Subsequent to Xenon Administration. Patients with only PD (group 1, n = 42) and “comorbid” PD (group 2, n = 39) where administered 6–7 sessions of xenon inhalation as described in “Methods”. Analysis of SAS score was performed. *Error bars* indicate 95% CI

Symptom changes on the SAS scale are presented in Fig. 2. The initial presentation in both groups corresponded to “high level of anxiety”(72.7 and 64.1, respectively). One month after treatment, all patients showed a decrease in the SAS total score, although it was more pronounced in group 1: 36.5 points (which corresponds to “no anxiety”) against 46.8 points in group 2 (“minimum degree of anxiety”). Furthermore, these parameters remained approximately at the same level throughout the study follow up (34.5 and 47.9, respectively). Statistical analysis of changes in SAS scale using paired test samples are presented in Table 5.

**Table 5:** Descriptive statistics for the Zung Self-Rating Anxiety Scale (SAS) and changes compared with baseline (V1) for the evaluation visits and patient groups

	Paired differences					t	df	Sig. (2-tailed)
	Mean	Std. deviation	Std. error mean	95% confidence interval of differences				
				Lower	Upper			
Group 1								
Pair 1								
Zung after 1 month, V1	−36.131	3.205	.495	−37.130	−35.132	−73.048	41	.000
Pair 2								
Zung after 6 months, V1	−38.214	4.049	.625	−39.476	−36.952	−61.158	41	.000
Group 2								
Pair 1								
Zung after 1 month, V1	−17.308	7.508	1.202	−19.742	−14.874	−14.395	38	.000
Pair 2								
Zung after 6 months, V1	−16.154	7.562	1.211	−18.605	−13.702	−13.340	38	.000

**Fig. 3:**
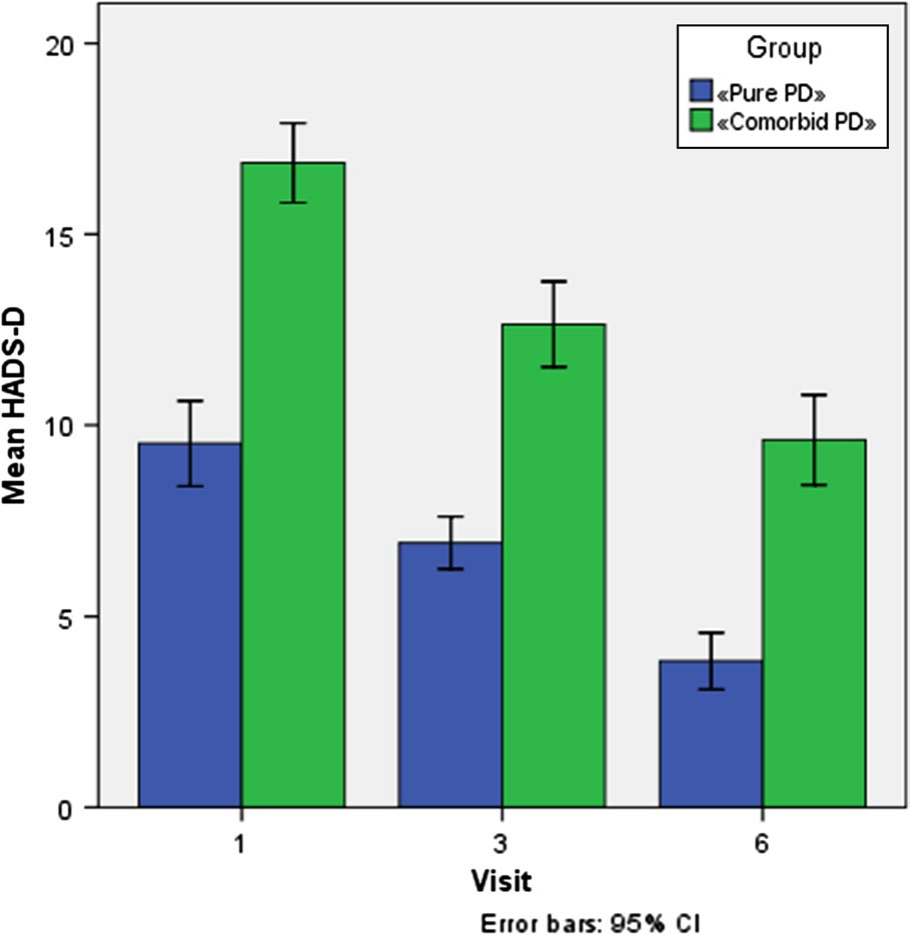
Changes in HADS D Scale (subscale “depression”) after Xenon Administration. Patients with only PD (group 1, n = 42) and “comorbid” PD (group 2, n = 39) where administered 6–7 sessions of xenon inhalation as described in “Methods”. Analysis of Analysis of HADS_T score was performed. *Error bars* indicate 95% CI

As noted above, in the modern classifications, in addition to “major” episodes that meet the criteria of a panic attack based on the number of symptoms, “limited symptom” (“minor”) panic attacks have been described, which, nevertheless, have an impact on social functioning and quality of life. As seen in Fig. 3, the mean number of “major” panic attacks per month in group 2 was even greater than that of the group 1 (7.7 and 11.7, respectively), while the number of “minor” attacks were slightly higher in group 1, or patients with “pure” PD (44.8 and 41.7, respectively). 6 months after treatment “major” panic attacks were absent in both groups, while “minor” panic attacks occurred in a very few cases (.3–1, respectively).

The results of SAS scales and lack of panic attacks after 6 months of treatment indicate the sustained anxiolytic effect of xenon administration.

As has already been noted, most often encountered comorbid mental conditions in group 2 included depressive disorders. The high degree of severity of depressive symptoms indicate that the traditional in these cases antidepressant therapy was ineffective. While the effect of xenon directly on depression is beyond the scope of this study, the analysis of HADS_T subscale “Depression” of HADS_D scale (Table 6) warrants some observations on this topic. According to HADS_D, “clinically severe depression” was absent in 66.7% of the patients in group 1 before the start of treatment, while it was present in 92.3% of group 2. After 3 xenon treatments it was absent in 90.5% of patients in group 1, but was still present in the majority of patients in group 2 (82.1%). By the end of the active phase of treatment “clinically severe depression” was negligible in patients of group 1 (2.4%), and it decreased to 46.2% in group 2.

**Table 6:** Changes in the frequency of major and minor panic attacks (per month)

	Group		
	“Pure” PD (n = 42)	“Comorbid” PD (n = 39)	Total (n = 81)
The frequency of major panic attacks before treatment, times/month, V1			
n	42	39	81
Mean	7.7	11.7	9.6
Standard deviation	7.85	8.27	8.24
Percentile 25	1.0	4.0	2.0
Median	3.0	12.0	7.0
Percentile 75	16.0	16.0	16.0
Minimum	1	1	1
Maximum	24	28	28
The frequency of major panic attacks 6 months after treatment			
n	42	39	81
Mean	.0	.0	.0
Standard deviation	.0	.0	.0
Percentile 25	.0	.0	.0
Median	.0	.0	.0
Percentile 75	.0	.0	.0
Minimum	0	0	0
Maximum	0	0	0
The frequency of minor panic attacks before treatment, times/month, V1			
n	42	39	81
Mean	44.8	41.7	43.3
Standard deviation	16.18	15.29	15.73
Percentile 25	28.0	28.0	28.0
Median	56.0	56.0	56.0
Percentile 75	56.0	56.0	56.0
Minimum	4	4	4
Maximum	84	56	84
The frequency of minor panic attacks 6 months after treatment, times/month			
n	42	39	81
Mean	.3	1.0	.6
Standard deviation	.46	2.64	1.89
Percentile 25	.0	.0	.0
Median	.0	.0	.0
Percentile 75	1.0	1.0	1.0
Minimum	0	0	0
Maximum	1	16	16
Changes minor panic attacks_V6_V1			
n	42	39	81
Mean	−44.5	−40.7	−42.7
Standard deviation	16.19	15.12	15.70
Percentile 25	−56.0	−56.0	−56.0
Median	−55.0	−40.0	−55.0
Percentile 75	−28.0	−27.0	−28.0
Minimum	−84.00	−56.00	−84.00
Maximum	−4.00	−4.00	−4.00
Changes_major panic attacks _V6_V1			
n	42	39	81
Mean	−7.7	−11.7	−9.6
Standard deviation	7.85	8.27	8.24
Percentile 25	−16.0	−16.0	−16.0
Median	−3.0	−12.0	−7.0
Percentile 75	−1.0	−4.0	−2.0
Minimum	−24.00	−28.00	−28.00
Maximum	−1.00	−1.00	−1.00

Xenon therapy was generally well tolerated, side effects, mainly headache and dizziness, were rare and lead to only 5 patients dropping out from the study (5.8%). After carefully reviewing the data from these patients, it should be noted that four of them were found to have clinical symptoms of mild organic brain disease of vascular origin (F06.71 heading ICD-10), which is indirectly confirmed by the results of head and neck Doppler ultrasound. It was previously demonstrated that inhalation of xenon can increase cerebral blood flow [6].

## Discussion

Despite the fact that SSRIs, SNRIs and benzodiazepines have proven efficacy in the treatment of PD, the delayed onset of action for the former and the side effects and the risk of dependence for the latter limit its use in the most active cohort of patients with PD. In addition, there are currently insufficient data on the treatment of refractory PD and on effective augmentation strategies, as well as on the treatment PD with comorbid mental illnesses.

The inert gas xenon was first shown to possess anesthetic properties over 50 years ago [43]. Over the last 10 years the interest in xenon as an inhalational anesthetic has increased due to several characteristics associated with its use: cardiovascular stability, rapid induction and emergence from anesthesia, and its analgesic effects—all of which make it an ideal anesthetic [44]. As a result, xenon has become more routinely used as an anesthetic agent in Europe and Japan and has garnered increasing interest in the United States although, primarily due to the higher cost of xenon as compared to other inhalational anesthetics, it has not yet received FDA approval. Evidence suggests that xenon’s biological effects may be mediated through its ability to potently block the NMDA receptors [45].

Furthermore, xenon has distinct advantages over other NMDA antagonists, such as ketamine, for future translation to the clinical setting. First, subsedative concentrations of xenon that would sufficiently block the NMDA receptor without producing anesthesia could potentially be administered briefly in a safe and effective manner in the outpatient setting with minimal medical monitoring. Second, in contrast to existing NMDA receptor blockers like ketamine, xenon has been shown to inhibit NMDA receptor activity through competitive inhibition of the co-agonist glycine at the glycine site of the NMDA receptor [45]—a mechanism devoid of psychotomimetic effects.

On the basis of the result of this study’s clinical and psychometric data with the use of scales assessing both the severity of anxiety (SAS, HADS_T), and evaluation of treatment effect in general (CGI-I, CGI-S), several preliminary conclusions can be made.

Firstly, when using xenon as an acute treatment of PD, reduction in both frequency and severity of panic attacks and anxiety level was observed during the first three treatment sessions, and by the end of treatment the vast number of patients experienced complete resolution of panic attacks while anxiety symptoms decreased to a subclinical level. This conclusion is also true for patients with PD with comorbidities. Secondly, after treatment of both “pure” PD and PD with psychiatric comorbidities, anxiolytic effect of xenon was maintained for at least 6 months, which clinically manifested by cessation of panic attacks. Thirdly, treatment with xenon was well tolerated, although it is possible that patients with symptoms of cerebral insufficiency may experience such adverse events as headache and dizziness. Fourthly, although the effect of xenon on depressive disorders was beyond the scope of this study, the analysis of the changes in HADS_D scale allows us to make a preliminary conclusion: in patients with PD and comorbid depressive symptoms xenon treatment can not only lead to improvement in anxiety symptoms, but also to reduction of depression severity. In this sense, we see as highly relevant a conduct of the study on the use of xenon as an adjuvant therapy for those patients with depressive disorders and concomitant anxiety (with anxious distress according to DSM-5), which have not sufficiently responded to treatment with conventional pharmacotherapy. Fifthly, the current first line of treatment of PD, in addition to pharmacotherapy, includes cognitive-behavioral therapy (CBT) [7]. At the same time, in clinical practice, the use of CBT in patients with severe PD in the early stages is often hampered by the high intensity of the somatic symptoms of anxiety and hypochondriacal fears, for relief of which providers often resort to the use of benzodiazepines. This approach can affect cognitive function and impede effective personal involvement in psychotherapy. In this sense, xenon treatment may be a good alternative to benzodiazepine tranquilizers because of its good tolerability, rapidity of onset and its lack of addictive potential.

The main methodological limitation of this study is its open design. In order to determine the place xenon in the treatment of PD additional randomized, placebo-controlled clinical trials of xenon and psychotropic substances used for the treatment of PD (SSRIs, SNRIs, benzodiazepines) are needed. The design of such studies should distinguish between direct anxiolytic effect of xenon and a potential placebo effect.

## Conclusions

The present study is the first work on the use of xenon in panic disorder that can give impetus to a more intensive research into xenon’s place in the treatment of anxiety and depressive disorders as both the adjunct and a potential alternative to the currently used psychotropic pharmacotherapy. Given the accepted use of subanesthetic concentrations of xenon in imaging, and established safety profile of concentrations similar to the ones utilized in the current study, future investigation of xenon based therapeutics in prospective double blind placebo controlled trials is warranted.

## Abbreviations

CGI-S: Clinical Global Impression Scale Severity of the Disease Subscale
HADS: Hospital Anxiety and Depression Scale
NDMA: *N*-methyl-d-aspartate
PD: panic disorder
SAS: Scale Assessment, Zung Self-Rating Anxiety Scale
SNRI: serotonin and norepinephrine reuptake inhibitor
SSRI: selective serotonin reuptake inhibitor
